# Health literacy strengths and challenges of people in New South Wales prisons: a cross-sectional survey using the Health Literacy Questionnaire (HLQ)

**DOI:** 10.1186/s12889-023-16464-3

**Published:** 2023-08-10

**Authors:** Scott Gill, Reem Zeki, Sharlene Kaye, Panayiota Zingirlis, Vicki Archer, Amy Lewandowski, Grantley Creighton, Caron Shaw, Julia Bowman

**Affiliations:** 1 Research Unit, Justice Health and Forensic Mental Health Network, Sydney, Australia; 2 Centre for Global Health and Equity, Swinburne University of Technology, Melbourne, Australia; 3 School of Medicine and Public Health, University of Newcastle, Callaghan, Australia; 4 National Drug and Alcohol Research Centre, University of New South Wales, Sydney, Australia; 5 Centre for Forensic Behavioural Science, Swinburne University of Technology, Melbourne, Australia; 6 Aboriginal Health, Justice Health and Forensic Mental Health Network, Sydney, Australia; 7 Adolescent Health, Justice Health and Forensic Mental Health Network, Sydney, Australia

**Keywords:** Health literacy, Health literacy questionnaire, HLQ, Health inequities, Prisons, Access, Public health

## Abstract

**Background:**

Health literacy is an important factor for enabling people to manage their health and live long fulfilling lives. People in prison are frequently from marginalised communities, often out of reach of conventional community based health organisations, and have poorer health outcomes. It is essential to understanding the health literacy profiles of people in prison, and its contribution to the well-established health inequities and outcomes of this population. This study aimed to use a multi-dimensional health literacy measurement tool to describe the strengths and challenges of adults incarcerated in NSW prisons.

**Methods:**

A cross-sectional survey was conducted for people in prison across 14 publicly operated metropolitan prisons. Data were collected from 471 participants using the Health Literacy Questionnaire (HLQ). Participant characteristics and health conditions were also collected. Data were analysed using descriptive statistics. Effect sizes (ES) for standardised differences in means were used to describe the magnitude of difference between participant characteristic groups.

**Results:**

Participants’ median age was 38.0 (range 19 – 91) years. Males comprised 81% of the sample, 21% identified as Aboriginal and/or Torres Strait Islander, and 53% reported a health problem. People in prison had lower scores for all nine HLQ scales when compared to the general Australian population. Small to medium ES were seen for mean differences between most demographic groups. Compared to males, females had lower scores for several of the HLQ scales including ‘having sufficient information to manage health’ (ES 0.30 [95% Confidence Interval (CI) 0.07, 0.53]), ‘ability to actively engage with health care professionals’ (ES 0.30 [95% CI 0.06, 0.53]), ‘navigating the healthcare system’ (ES 0.30 [95% CI 0.06, 0.53]), and, ‘ability to find good health information’ (ES 0.33 [95% CI 0.10, 0.57]). Differing health literacy scale scores with small to medium ES were found when comparing participants by legal status. Mainly small ES were seen when comparing other participant characteristic groups.

**Conclusions:**

This study provides insights into the health literacy strengths and challenges for people in NSW prisons. These findings highlight the important role health literacy could have in addressing health disparities in this vulnerable population and can inform prison health services.

## Background

### The health of people in prison

People in a prison are often forgotten and marginalised members of our society, hidden out of the eyes of the general public. Over the past two decades, the global prison population has grown to over 10.77 million, an increase of 24 per cent [1]. Australia is not immune to this phenomenon, with the prison population growing 98 per cent for the same period [1]. As of 30 September 2022, 40,907 adults were incarcerated in Australia, and 12,467 in the state of New South Wales (NSW) [2]. Like other Australian state and territory jurisdictions, the NSW prison population has a vast overrepresentation of people who identify as Aboriginal and/ or Torres Strait Islander (hereafter, respectfully referred to as Aboriginal people). At the aforementioned date, Aboriginal people accounted for 28 per cent of the incarcerated adult population in NSW prisons [2], whilst only representing approximately 3.4 per cent of the general NSW population [3].

Adults who are incarcerated frequently have complex health and social needs. Research has consistently shown that those in contact with the criminal justice system have poorer health outcomes, a high prevalence of health related diseases [4–8], higher rates of unemployment and lower formal education levels [9, 10]. More specifically, people in contact with the criminal justice system in Australia have higher rates of mental health conditions, and both communicable and non-communicable diseases when compared to the general Australian population [9–11].

In Australia, services providing healthcare in prisons vary between jurisdictions. In NSW, the Justice Health and Forensic Mental Health Network (Justice Health NSW) provides health care to individuals in custody within publicly run prisons. Justice Health NSW is bound by the widely accepted Nelson Mandela Rules [12]. These rules task governments and prison authorities with ensuring that people in prison have the right to adequate health care, equivalent to those who are not incarcerated. Authorities should, however, be aiming to achieve equivalent health outcomes within this population, not just equivalent health care [13]. In order to achieve equivalent health outcomes, researchers, policy makers and relevant authorities need to ensure health care services are equitable (e.g., not a one-size fits all approach) for those who are in prison.

Prisons are an important setting in which to access vulnerable individuals who are out of the reach of conventional community based health organisations [4]. Prison health care providers have the opportunity to deliver evidenced based health care such as screening, preventive and chronic care to a vulnerable group of our society [6]. Whilst someone is in contact with the criminal justice system, there is an opportunity to promote health and teach self-management skills to this marginalised population [8, 14]. Therefore, interventions delivered in custody provide the opportunity for people in prison to increase their knowledge and understanding of health; and build their capability to self-manage their health both whilst incarcerated and upon release to the community.

### Health literacy and its potential

In addition to the accessibility and equity of health care, health literacy is an important factor contributing to differential health outcomes, with health literacy recently described as a modifiable social determinant of health [15]. Health literacy is an evolving concept, repeatedly being defined and redefined by academics globally [16–19]. Common misconceptions about health literacy include that it relates only to the functional literacy and numeracy skills of an individual. However, health literacy refers to how an individual and their surrounding community access health information, engage with health services, and manage and make informed decisions about their health [20]. Furthermore, health literacy represents how health information and services are utilised by people to maintain and promote good health for themselves and others and is thus mediated by the resources accessible and the structures of organisations providing health care [20]. It has been previously reported that between 25 per cent and 60 per cent of the general Australian population has “low” health literacy [21, 22]. “Lower” health literacy has been associated with poorer health outcomes, poorer health services utilisation [23], lower educational levels and lower socio-economic backgrounds [24, 25], all of which are prevalent in the NSW prison population. This highlights the important role health authorities’ play when providing care, particularly to vulnerable and marginalised populations.

The World Health Organization (WHO) has redefined and refocused health literacy to represent the series of decisions (sometimes complex) made by a person when trying to access, understand and use information to make decisions about their health [20], thereby shifting the focus of health literacy from being solely an individual’s experience, to also include their community and the health literacy responsiveness of organisations. That is, the extent to which health literacy strengths and challenges are identified and accommodated to facilitate access to, and engagement with health information and services. When referring to health literacy strengths and challenges, we mean, taking a strengths-based approach to health literacy measurement and considering contextual and cultural norms of the specific contexts (i.e., prisons). When describing a person's strengths in health literacy, it refers to their skills and abilities to be able to find, understand, and use information and services to inform health-related decisions and actions for themselves and others. High levels of social support, for example would indicate that a strength for them in making health-related decisions is being supported by their social connections. Conversely, their challenges reflect things that make it difficult to do this. They may have limited understanding of health information and, therefore, would require support in being able to make health-related decisions from this information. In order for a community or organisation to become responsive to health literacy, the important first step is to measure and describe the health literacy of the individuals to which health services are provided.

There are a large number of tools to measure health literacy [26, 27], with most contemporary tools focusing on the functional literacy and numeracy of individuals [28]. Other health literacy tools measure the concept in relation to a specific disease [e.g. Diabetes Numeracy Test [29] and Cancer Health Literacy Scale [30]]. The specificity of these measurement tools limits their application in large non-homogenous populations as found within prisons [28]. Further, most health literacy measurement tools produce an overall and “cut-off” scores [31], minimising and reducing the complexity of health literacy to a singular number [32]. The singular number is then utilised to classify individuals, using “cut-off” scores, as having low, medium or high health literacy, which does not reflect the real world experience of an individual [31]. To respond to the evolving concept of health literacy, the Health Literacy Questionnaire (HLQ) [33] was developed in 2013 through a grounded and validity-driven approach to capture a realistic profile of an individual’s or a community’s health literacy strengths and, challenges [33]. Given the increasing recognition and growing evidence base of the importance of health literacy and how this may positively impact an individual’s or population’s collective skills to manage health, it is important to investigate this within prison populations.

To the authors’ knowledge, no other study has utilised the HLQ in a prison context. Only one other study [34] has recently investigated the health literacy of adults who are incarcerated using a multidimensional tool. However, it has limited application to the broader prison population and in particular the Australian prison context due to the modifications made to the original measurement tool (i.e., European Health Literacy Survey Questionnaire [HLS-EU-Q] [35]) and the limitations noted by the authors [34]. That is, the study was conducted in a single English prison, among young male participants (18–21 years), limiting the generalisability of findings beyond the study setting [34].

This study aimed to measure and describe the health literacy profile of a sample of adults who were incarcerated in NSW and have accessed the health services available in metropolitan correctional centres.

## Methods

### Study design

The *2021 Health Literacy Study: People in NSW Prisons and a High Secure Forensic Setting* (2021 Health Literacy Study) was a cross-sectional survey that investigated the health literacy of adults in secure settings (i.e., correctional centres and a high secure psychiatric hospital) in NSW. This paper describes the findings from the survey for the prison context.

### Data source

Cross-sectional data were collected as part of the 2021 Health Literacy Study, a patient survey conducted by Justice Health NSW which aimed to investigate the health literacy of adults in secure settings in NSW. Full details of the methods have been published elsewhere [36]. Justice Health NSW is a statutory health corporation and part of the greater NSW Health public system which provides health care to people in contact with the criminal justice and forensic mental health systems. It provides a wide range of nursing, medical and allied health services including Aboriginal Health, Mental Health, Drug and Alcohol programs, Population Health, Oral Health, Primary Health, and Women’s Health. People in prison are referred to Justice Health NSW services through channels, such as self-referral, clinician-referral based on reception screening or escalation pathways, routine screening and monitoring of known health conditions. Services offered by Justice Health NSW vary between each healthcare setting. The services provided are similar to community out-patient clinics, with mainly primary and limited secondary healthcare services provided [37]. Further, if people in prison require acute care, they are transferred to a Local Health District equipped to provide the necessary care.

### Study eligibility and sampling

The sample for this study comprised adults who were in the custody (sentenced or on remand) of Corrective Services New South Wales (CSNSW) during the survey period (October 2020—April 2021). Participant eligibility criteria included the participant’s ability to speak and comprehend English, consent to the study procedures and that they had used the prison primary health care nursing service between 1 October 2019 and 31 September 2020.

Participants were recruited based on a stratified random sample design. Sample size calculations per correctional centre health clinic were calculated utilising a finite proportion calculation as described by Israel [38]. Sample size calculations were stratified by health centre in the correctional centres and Aboriginal identity (25%). Stratification by Aboriginal identity at 25 per cent was undertaken to ensure adequate representation of Aboriginal people in the participant sample, as at 30 June 2020 Aboriginal people made up over one quarter (25.1%) of the NSW prison population [39]. The full details of the sample size calculations and recruitment methods are reported elsewhere [36].

### Data collection

The survey was conducted at 14 correctional centres. Data were collected from participants through structured face-to-face interviews. Interviews were conducted in prison health clinics, wings and general visit areas. Participants were verbally administered the HLQ by a trained interviewer. Self-reported demographic (level of secondary and tertiary education attained, primary language spoken at home, identification as Aboriginal and/or Torres Strait Islander) and health status (perceived health rating and presence of a health condition) data were collected directly from participants. An electronic platform (Qualtrics) [40] was used for data collection. Where the use of Qualtrics was not possible, paper-based recording of data was used, with data subsequently entered into the Qualtrics platform. Routinely collected custodial (location, security classification, correctional sentence and sentence length) and demographic (age and sex) data were extracted from the Offender Integrated Management System and Patient Administration System, managed by CSNSW and Justice Health NSW, respectively.

### The Health Literacy Questionnaire

The HLQ is a comprehensive 44-item health literacy measurement tool, which captures the latent multi-dimensional concept of health literacy across nine independent albeit complimentary scales [33]. As described by Osborne et al. [33] the nine scales of the HLQ are:

1. Feeling understood and supported by healthcare providers;
2. Having sufficient information to manage my health;
3. Actively managing my health;
4. Social support for health;
5. Appraisal of health information;
6. Ability to actively engage with healthcare providers;
7. Navigating the healthcare system;
8. Ability to find good health information;
9. Understand health information enough to know what to do.

Each scale of the HLQ has between 4 and 6 items that are scored on a Likert scale. There are four response options for the items in scales 1–5: *strongly disagree, disagree, agree* and *strongly agree*. The last 4 scales (scales 6–9) have five response options for the items: *cannot do or always difficult, usually difficult, sometimes difficult, usually easy* or *always easy*. Scores for each scale are calculated by summing item scores and dividing by the number of items within the scale [33]. A total score is not calculated for the nine HLQ scales. Mean scale scores are calculated and interpreted separately for each scale [33]. Scale scores range between 1 and 4 for scales 1 to 5, and 1 and 5 for the last four scales.

The HLQ was developed through a grounded and validity-driven approach [33]. The HLQ has been translated into more than 30 languages and validity evidence has shown each scale to be highly reliable across a diverse range of contexts globally [33, 41–56]. Despite the vast amount of validity evidence gathered for the HLQ, this study is the first to use the multi-dimensional tool in a prison context.

### Statistical analysis

HLQ scale scores and participant characteristic data were analysed using IBM SPSS Version 27 [57]. Two participants who completed informed consent processes were excluded from data analysis due to missing a greater number of items than could be imputed from further analyses. Findings in this article have been weighted to account for the over-representation of both non-Aboriginal people and females in the recruited sample. The weighting ensured that findings for the total population, those who identified as Aboriginal and females reflect all data gathered but avoid the potential for bias by disproportionate numbers of participants in specific demographic groups. Weighting calculations have been reported elsewhere [36].

For all HLQ scales, responses covered the full range of the scale with no apparent floor or ceiling effects and the assumptions of normal distribution were met. Therefore, we utilised independent t-tests for analysis of HLQ scores for dichotomous variables and robust analysis of variance (ANOVA) using Welch’s method for categorical variables [58]. The Games-Howell method of multiple mean comparisons was undertaken where required. Effect sizes (ES) for standardised differences in means between dichotomous participant demographic groups were calculated using Cohen’s d (calculated as the difference between the two means, divided by the pooled standard deviation (SD) of both means). The interpretation of ES were as follows: small (d = 0.2), medium (d = 0.5), and large (d = 0.8) [59]. ES for standardised average effect across variable means between categorical groups were calculated using Omega-squared $\left({\omega }^{2}\right)$. The interpretation of ${\omega }^{2}$ were as follows: small $\left({\omega }^{2}=0.01\right)$, medium $\left({\omega }^{2}=0.06\right)$ and large $\left({\omega }^{2}=0.14\right)$ [60]. Cronbach’s alpha was calculated to assess the internal consistency of the nine HLQ scales. The interpretation of Cronbach’s alpha with regard to level of consistency was as follows: questionable (≥ 0.60 to 0.69) and acceptable (≥ 0.70 to 0.95) [61, 62]. Where relevant, 95% confidence intervals were calculated. A p-value of < 0.05 was assumed for statistical significance.

### Ethics

Human Research Ethics Committee (HREC) approval was obtained from the Justice Health NSW HREC (2019/ETH00415), Aboriginal Health and Medical Research Council HREC (1664/20) and CSNSW Ethics Committee (DG20/001384). Informed written consent was obtained from all participants. The informed written consent process for participants is described in further detail elsewhere [36].

## Results

### Participant characteristics

A total of 471 participants were recruited from across 14 correctional centres, ranging from 19 to 44 participants per site. The median (SD) age of participants was 38.0 (13.9) years, ranging from 19 to 91 years. Males compromised 81.3% of the participant sample. Just over one fifth (20.6%) self-identified as Aboriginal, Torres Strait Islander or both. Over half (53.3%) of participants reported the presence of a health condition. Over two thirds (68.2%) of participants were currently serving a custodial sentence, with the remainder on remand. Under one third (31.2%) reported they had completed Year 12. The majority (86.2%) stated they spoke primarily English at home. Participant characteristic data are shown in Table 1.

**Table 1:** Participant characteristic data for overall sample (*n* = 471)

	**n**	**(%)**	**Don't Know/Not Stated (n)**
Male	383	81.3	0
Age ≥ 45 years	169	35.9	0
Identifies as Aboriginal/Torres Strait Islander	97	20.6	0
English spoken at home	406	86.2	1
Completed Year 10 or lower	280	59.4	7
Completed High School	147	31.2	22
Post-high school qualification	156	33.1	0
Sentenced	321	68.2	0
Security Classification—Max	181	38.4	0
Security Classification—Minimum	205	43.5	0
Reports no health conditions	220	46.7	4

### Health Literacy Questionnaire scores

Mean scores for each HLQ scale for people in prison and the general Australian population are shown in Table 2. Compared to the general Australian population [63], people in prison had statistically significant and lower mean scores for all nine HLQ scales. For the first five scales of people in prison, the highest overall score was seen for the scale 3—Actively managing my health (mean score 3.03 [SD 0.45]). The lowest score was for scale 5—Appraisal of health information (mean score 2.57 [SD 0.49]). For the last four scales, the highest overall score was seen for scale 9—Understand health information enough to know what to do (mean score 4.00 [SD 0.70]). The lowest scale score was seen for 7—Navigating the healthcare system (mean score 3.11 [SD 0.87]).

**Table 2:** Health Literacy Questionnaire (HLQ) scores for the overall sample and General Australian Population [63]

	**People in Prison**		**General Australian Population**
	Cronbach’s Alpha	Mean (SD) [95% CI]	Mean (SD) [95% CI]
HLQ scale			
		Range 1 (lowest) - 4 (highest)	
1. Feeling understood and supported by healthcare professionals	0.81	2.69* (0.58) [2.64–2.74]	3.18 (0.77) [3.16–3.20]
2. Having sufficient information to manage my health	0.79	2.73* (0.51) [2.68–2.78]	3.17 (0.63) [3.15–3.19]
3. Actively managing my health	0.83	3.03* (0.45) [2.99–3.07]	3.09 (0.68) [3.07–3.11]
4. Social support for health	0.69	2.59* (0.50) [2.54–3.07]	3.19 (0.70) [3.17–3.21]
5. Appraisal of health information	0.76	2.57* (0.49) [2.54–3.07]	2.92 (0.67) [2.90–2.94]
		Range 1 (lowest) - 5 (highest)	
6. Ability to actively engage with healthcare professionals	0.88	3.37* (0.89) [3.29–3.45]	4.18 (0.86) [4.16–4.20]
7. Navigating the healthcare system	0.87	3.11* (0.87) [3.03–3.19]	4.02 (1.01) [3.99–4.05]
8. Ability to find good health information	0.82	3.13* (0.84) [3.05–3.20]	4.09 (0.90) [4.07–4.11]
9. Understand health information enough to know what to do	0.78	4.00* (0.70) [3.94–4.06]	4.27 (0.94) [4.25–4.29]

Results in bold with * have *p*-value < 0.05 for difference in means (tested using independent t-test). *Abbreviations*: *SD* Standard Deviation, *95% CI* Confidence Interval

Overall, the internal consistency of the nine HLQ scales was found to be relatively high, ranging from 0.69 to 0.88 (Table 2). For the first five scales the highest internal consistency was found for Scale 3—Actively managing my health (0.83). The lowest was found for scale 4 – Social support for health (0.69). For the last four scales, the highest internal consistency was found for scale 6 – Ability to actively engage with healthcare professionals (0.88). The lowest was found for scale 9 – Understand health information enough to know what to do (0.78). Thus, eight of the nine scales were found to have acceptable internal consistency, with scale 4 having a level of consistency at the upper limit of the ‘questionable’ alpha range.

Table 3 shows patterns of HLQ scale scores according to demographic characteristics.

**Table 3:** Association between HLQ scores and participant characteristics

			1. Feel understood & supported by health care professionals	2. Have sufficient information to manage health	3. Actively managing my health	4. Social support for health	5. Appraisal of health information	6. Ability to actively engage with health care professionals	7. Navigating the Healthcare System	8. Ability to find good health information	9. Understand health information
		n	Mean score (SD)								
Sex	Male	383	2.69 (0.58)	**2.74 (0.51)**	3.03 (0.45)	2.59 (0.50)	2.57 (0.49)	**3.39 (0.87)**	**3.13 (0.86)**	**3.14 (0.83)**	4.00 (0.70)
	Female	88	2.73 (0.61)	2.59 (0.56)*	3.00 (0.50)	2.56 (0.55)	2.59 (0.48)	3.12 (1.02)*	2.87 (0.96)*	2.86 (0.89)*	3.97 (0.68)
Effect size for sex (95% CI)			-0.07 (0.30, 0.17)	0.30 (0.07, 0.53)	0.06 (-0.17, 0.29)	0.05 (0.18, 0.28)	-0.03 (-0.26, 0.20)	0.30 (0.06, 0.53)	0.30 (0.06, 0.53)	0.33 (0.10, 0.57)	0.04 (-0.19, 0.27)
Aboriginal Identity	No	375	2.70 (0.56)	2.72 (0.52)	3.05 (0.46)	2.60 (0.51)	2.59 (0.50)	3.38 (0.88)	3.08 (0.85)	3.13 (0.83)	**4.05 (0.67)**
	Yes	97	2.66 (0.62)	2.77 (0.50)	2.97 (0.44)	2.60 (0.50)	2.54 (0.45)	3.33 (0.92)	3.19 (0.91)	3.11 (0.87)	3.86 (0.78)*
Effect size for Aboriginal Identity (95% CI)			0.07 (-0.15, 0.30)	-0.09 (-0.32, 0.13)	0.17 (-0.06, 0.39)	0.08 (-0.15, 0.30)	0.11 (-0.12, 0.33)	0.05 (-0.17, 0.28)	-0.13 (-0.35, 0.10)	0.03 (-0.20, 0.25)	0.26 (0.04, 0.49)
Age group	< 45 yr	300	2.67 (0.58)	2.74 (0.52)	3.04 (0.46)	2.59 (0.50)	2.55 (0.47)	**3.29 (0.88)**	3.09 (0.85)	3.08 (0.82)	**3.95 (0.68)**
	≥ 45 yr	172	2.74 (0.57)	2.72 (0.50)	3.01 (0.44)	2.59 (0.52)	2.62 (0.51)	3.51 (0.88)*	3.15 (0.89)	3.21 (0.86)	4.09 (0.72)*
Effect size age (95% CI)			-0.13 (-0.32, 0.06)	0.03 (-0.16, 0.22)	0.06 (-0.12, 0.25)	-0.01 (-0.20, 0.18)	-0.14 (-0.33, 0.55)	-0.26 (-0.45, -0.07)	-0.08 (-0.26, 0.11)	-0.15 (-0.34, 0.04)	-0.20 (-0.39, -0.01)
Completed above year 10	No	288	2.68 (0.59)	2.74 (0.50)	**2.98 (0.45)**	2.60 (0.50)	2.56 (0.49)	3.38 (0.89)	3.15 (0.87)	3.15 (0.87)	**3.94 (0.68)**
	Yes	175	2.71 (0.58)	2.71 (0.54)	3.11 (0.46)*	2.56 (0.52)	2.59 (0.48)	3.35 (0.88)	3.04 (0.85)	3.08 (0.83)	4.13 (0.67)*
Effect size education (95% CI)			-0.05 (-0.24, 0.13)	0.06 (-0.13, 0.25)	-0.28 (-0.46, -0.09)	0.08 (-0.11, 0.27)	-0.08 (-0.26, 0.11)	0.04 (-0.15, 0.22)	0.13 (-0.06, 0.31)	0.09 (-0.10, 0.28)	-0.27 (-0.46, -0.09)
Highest education attainment	Secondary	308	2.70 (0.57)	2.75 (0.51)	3.02 (0.44)	2.61 (0.50)	2.58 (0.48)	3.37 (0.88)	**3.16 (0.88)**	3.17 (0.84)	**3.92 (0.70)**
	Tertiary	147	2.67 (0.62)	2.68 (0.53)	3.07 (0.48)	2.54 (0.53)	2.54 (0.52)	3.33 (0.88)	2.96 (0.80)*	3.01 (0.81)	4.21 (0.57)*
Effect size educational attainment (95%, CI)			0.04 (-0.16, 0.23)	0.15 (-0.05, 0.34)	-0.12 (-0.32, 0.07)	0.14 (-0.06, 0.33)	0.10 (-0.10, 0.29)	0.05 (-0.15, 0.25)	0.24 (0.04, 0.43)	0.19 (-0.01, 0.38)	-0.44 (-0.64, -0.24)
Legal status	Sentenced	320	**2.74 (0.58)**	**2.76 (0.50)**	3.02 (0.45)	2.61 (0.52)	2.60 (0.50)	3.41 (0.88)	3.15 (0.84)	3.15 (0.81)	4.02 (0.66)
	Remand	151	2.58 (0.55)*	2.66 (0.53)*	3.05 (0.46)	2.55 (0.47)	2.52 (0.47)	3.29 (0.88)	3.02 (0.91)	3.08 (0.89)	3.95 (0.77)
Effect size Status (95%, CI)			0.27 (0.08, 0.47)	0.20 (0.00, 0.39)	-0.06 (-0.25, 0.13)	0.11 (-0.08, 0.30)	0.17 (-0.02, 0.36)	0.13 (-0.06, 0.33)	0.15 (-0.04, 0.35)	0.08 (-0.11, 0.28)	0.10 (-0.10, 0.29)
English spoken at home	No	57	2.62 (0.54)	2.71 (0.49)	3.12 (0.39)	2.59 (0.48)	2.65 (0.44)	3.31 (0.91)	3.01 (0.80)	3.03 (0.76)	3.94 (0.64)
	Yes	413	2.70 (0.58)	2.73 (0.52)	3.01 (0.46)	2.59 (0.51)	2.57 (0.49)	3.37 (0.88)	3.12 (0.87)	3.14 (0.85)	4.00 (0.71)
Effect size English (95%, CI)			0.08 (-0.19, 0.36)	0.04 (-0.23, 0.32)	-0.24 (-0.52, 0.03)	-0.01 (-0.29, 0.27)	-0.18 (-0.46, 0.10)	0.08 (-0.20, 0.36)	0.12 (-0.15, 0.40)	0.13 (-0.15, 0.41)	0.09 (-0.19, 0.36)
Prevalence of health condition	No	224	**2.62 (0.57)**	2.76 (0.50)	3.07 (0.41)	2.62 (0.48)	2.59 (0.47)	3.39 (0.87)	3.13 (0.83)	3.19 (0.82)	3.99 (0.71)
	Yes	244	2.76 (0.58)*	2.70 (0.52)	3.00 (0.49)	2.57 (0.52)	2.56 (0.51)	3.36 (0.90)	3.04 (0.89)	3.07 (0.85)	4.02 (0.69
Effect size condition (95% CI)			0.23 (0.05, 0.41)	-0.12 (-0.30, 0.06)	-0.15 (-0.33, 0.03)	-0.10 (-0.28, 0.08)	-0.05 (-0.24, 0.13)	-0.04 (-0.22, 0.14)	-0.17 (-0.35, 0.01)	-0.14 (-0.32, 0.04)	0.04 (-0.14, 0.23)
Perceived health rating	Very Good/Good	260	2.75 (0.55)	**2.84 (0.50)^**	**3.15 (0.42)^**	**2.69 (0.47)^**	**2.63 (0.48)^**	**3.51 (0.83)^**	**3.23 (0.83)^**	**3.27 (0.82)^**	**4.09 (0.64)^**
	Fair	157	2.63 (0.60)	**2.64 (0.48)**^**#**^	**2.89 (0.45)**^**#**^	**2.51 (0.51)**^**#**^	2.53 (0.46)	**3.27 (0.87)**^**#**^	**2.98 (0.83)**^**#**^	**3.00 (0.80)**^**#**^	**3.91 (0.69)**^**#**^
	Poor/Very Poor	53	2.59 (0.64)	**2.50 (0.50)**^**#**^	**2.81 (0.44)**^**#**^	**2.36 (0.53)**^**#**^	2.45 (0.55)	**3.01 (1.01)**^**#**^	**2.67 (0.91)**^**#**^	**2.82 (0.87)**^**#**^	3.85 (0.92)
Effect size health rating (95% CI)			0.01 (0.00, 0.03)	0.05 (0.02, 0.10)	0.10 (0.05, 0.15)	0.05 (0.02, 0.09)	0.01 (0.00, 0.04)	0.03 (0.01, 0.07)	0.06 (0.02, 0.10)	0.04 (0.01, 0.07)	0.02 (0.00, 0.04)

Results in bold with “*” have *p*-value < 0.05 for difference in means (tested using independent t-test); results in bold with “^” have *p*-value < 0.05 for an overall between-group difference in means (tested using robust ANOVA); results with “^#^” have *p*-value < 0.05 for difference in means to reference variable (Very Good/Good; tested using Post Hoc Games-Howell). Abbreviations Effect Size = ES. Dichotomous variables ES calculated using Cohen’s d for standardised difference in means. Interpretation of Cohen’s d ES: “small” ES > 0.20–0.50 SD, “medium” ES approximately 0.50–0.80 SD, and “large” ES > 0.80 SD. Categorical variables ES calculated using Omega-squared $\left({\omega }^{2}\right)$ for standardised average effect across variable means. Interpretation of ${\omega }^{2}$ ES: "small" ES > 0.01–0.05, "medium" ES 0.06–0.13, and "large" ES > 0.13

### Health literacy in specific participant characteristic groups

The largest effect sizes for differences between means were seen between male and female participants. Female participants had lower scores across seven of the nine HLQ scales when compared to males. Small to medium effect sizes for statistically significant differences between means were found for scales: 2—Having sufficient information to manage health (ES = 0.30 [0.07, 0.53]), 6—Ability to actively engage with healthcare professionals (ES = 0.30 [0.06, 0.53]), 7—Navigating the healthcare system (ES = 0.30 [0.06, 0.53]) and 8—Ability to find good health information (ES = 0.33 [0.10, 0.57]). Small and non-significant effect sizes were seen between means for the remaining scales.

Similar HLQ scale scores, with small ES, were seen when comparing participants who identified as Aboriginal to those who did not. Participants who identified as Aboriginal had lower scores for six of the HLQ scales when compared to non-Aboriginal participants: 1—Feeling understood and supported by healthcare providers (ES = 0.07 [-0.15, 0.30]), 3—Actively managing my health (ES = 0.17 [-0.06, 0.39]), 5—Appraisal of health information (ES = 0.11 [-0.12, 0.33]), 6—Ability to actively engage with healthcare providers (ES = 0.05 [-0.17, 0.28]), 8—Ability to find good health information (ES = 0.03 [-0.20, 0.25]) and 9—Understand health information well enough to know what to do (ES = 0.26 [0.04, 0.49]), which was the only significant mean difference.

Participants who reported having a health condition had higher scores for two of the HLQ scales when compared to their counterparts without a health condition for 1—Feeling understood and supported by healthcare providers (ES = 0.23 [0.05, 0.41]) and 9—Understand health information well enough to know what to do (ES = 0.04 (-0.14, 0.23]), although the effect sizes were small and only scale 1 scores differed significantly. Participants who perceived their health to be very good or good had higher mean HLQ scale scores for all nine HLQ scales compared to those who did not perceive their health as highly (i.e., fair and poor/very poor). Significant differences between groups were observed across all scales, except for scale 1—Feeling understood and supported by healthcare providers, with small to medium effect sizes $\left({\upomega }^{2}\mathrm{range}=0.01\;\mathrm{ to }\;0.10\right)$. Post hoc comparisons revealed significant differences between very good/good and both fair and poor/very poor for six of the nine HLQ scales. A significant difference was observed for Scale 9—Understand health information well enough to know what to do when comparing those who perceive their health to be very good or good to those who rated it as fair.

Older participants (older than 45 years) reported significantly greater abilities on scales 6—Ability to actively engage with healthcare providers (ES = -0.26 [-0.45. -0.07]) and 9—Understand health information well enough to know what to do (ES = -0.20 [-0.39, -0.01]) than younger participants; however, effect sizes were small. Participants who did not speak English at home were more likely to report greater abilities on scales 3- Actively managing my health (ES = -0.24 [-0.52, 0.03]) and 5—Appraisal of health information (ES = -0.18 [-0.46, 0.10]) than English speaking participants, although these differences were non-significant with small effect sizes.

### Health literacy by educational attainment

Small to medium effect sizes were seen for differences in HLQ scale scores according to educational attainment. The largest effect size was observed between participants who had completed tertiary education and those who had not completed education beyond high school, with significant differences across two of the nine HLQ scales (7—Navigating the healthcare system ES = 0.24 [0.04, 0.43]; 9—Understanding health information enough to know what to do (ES = -0.44 [-0.64, -0.24]). Although those who had completed tertiary education had higher scores for scale 9—Understanding health information enough to know what to do (ES = -0.44 [-0.64, -0.24]), they had lower scores for scale 7 – Navigating the healthcare system (ES = 0.24 [0.04, 0.43]). Participants who had completed Year 11 or above had significantly higher scores than their less educated peers (Year 10 or below) for scales 3—Actively managing my health (ES = -0.28 [-0.46, -0.09]), and 9—Understanding health information enough to know what to do (ES = -0.27 [-0.46, -0.09]).

### Health literacy challenges of those in different prison contexts

Participants who were sentenced had higher scores than those on remand for eight of the nine HLQ scales, with small to medium effect sizes. The only significant differences, and those with the largest effect sizes, were for scores on scales 1—Feeling understood and supported by healthcare providers (ES = 0.27 [0.08, 0.47]) and 2—Having sufficient information to manage my health (ES = 0.20 [0.004, 0.39]).

## Discussion

Health literacy was measured using the HLQ, to help identify people’s strengths and challenges in being able to access, understand and utilise health information and health services in prison. The results of this study are novel and provide a detailed picture of the health literacy profiles of people incarcerated in NSW metropolitan prisons. This study demonstrates that people in NSW metropolitan prisons have lower health literacy scores when compared to the general Australian population. Furthermore, small to medium differences in health literacy scores were observed within participant characteristic groups of people in NSW prisons. Particular groups with the largest health literacy differentials compared to their counterparts were females, those with lower education levels, and those who were on remand. Differences were also observed according to Aboriginal identity, age group, presence of a health condition, self-perceived health ratings and English spoken at home. It is important to note that although the effect sizes are only medium to small in magnitude, these differences may have compounding effects on the health outcomes of this specific group.

### Lower health literacy among marginalised populations

People in prison are well known to be a marginalised population in terms of health inequalities [4–8]. In our study, the overall participant sample had significantly lower health literacy scores when compared to the general Australian population. The participants reported some degree of difficulty across all nine scales of the HLQ. This finding reaffirms that people in prison are marginalised members of the community who have difficulty accessing and engaging with health care systems. Participant characteristic groups within the sample with lower health literacy scores include females and those who were on remand. These groups reported difficulties accessing and understanding health information, engaging with and feeling understood by healthcare providers and navigating the healthcare system. Such population groups (i.e., females) in the prison environment are known to be disadvantaged in terms of health outcomes [6, 64], with these disparities having been attributed, at least in part, by health literacy challenges in the low scoring scales identified in this study. Therefore, this study highlights that there is a significant amount of work to be done to address the identified health literacy strengths and challenges of those who are incarcerated. Furthermore, the health services provided to those in prison need to be tailored and equitable to suit specific participant characteristic groups within the population, rather than based on the current “one-size fits all” approach, to reduce the observed health and health literacy disparities of participant characteristic groups.

### Health literacy in specific participant characteristic groups

The prison population is non-homogenous and consistently in a state of change with people entering and leaving custody. Despite the ever-changing population, specific demographic groups within the incarcerated population do exist.

Females scored significantly lower than their male counterparts on four of nine health literacy scales; specifically, the scales that focus on engaging with healthcare providers, navigation of the healthcare system, and finding or possessing sufficient health information. Our finding is converse to the HLQ scores for males and females in the general Australian population, where females had higher scores across all nine scales [63]. The reasons for this finding are complex and may be due to many contributing factors. Females in prison are some of the most vulnerable members of society. They are disconnected from health care services and prevention information and access [65], have a higher burden of chronic medical conditions [6] and significant social deprivation, and abuse (i.e., substance and physical) and trauma histories [66–69]. Furthermore, females are placed in a male-dominated criminal justice system [70] which can restrict access to programs and access to specific women’s health services [67–69]. Therefore, our findings highlight the need for targeted policies and services for females in prison as addressed in the literature [6, 65, 66, 69, 71]. However, further research is needed to investigate the specific health literacy challenges of females and males in prison.

People in prison who identified as Aboriginal reported similar health literacy scores for a majority of the HLQ scales when compared with non-Aboriginal people in prison, with mainly small effect sizes observed between groups. The greatest and only significant difference between Aboriginal and non-Aboriginal people in prison was found for the scale 9—Understanding health information well enough to know what to do. This finding may be in relation to the cultural differences that exist between Aboriginal and non-Aboriginal people. For example, a study undertaken by Carroll et al. [72] investigating medication knowledge suggested that differences between Aboriginal and non-Aboriginal participants, which remained after adjusting for social disadvantage factors, could be related to cultural factors. Furthermore, Aboriginal people in prison have limited access to culturally appropriate health care which may influence their engagement with health information and the broader health system [73]. Similar scores were observed when comparing Aboriginal and non-Aboriginal participants for the remaining health literacy scales. The similarity suggests that regardless of cultural or ethnic background, socio-economic disadvantage (e.g., low educational attainment and unemployment) is a major determinant of people in prison experiencing vulnerabilities, such as poor health [74]. Despite these results, authorities need to focus on providing culturally responsive programs for specific groups in this context [75–77].

Younger participants (< 45 years of age) reported more difficulties with actively engaging with health care professionals and understanding health information. The reported difficulties in these two scales by younger participants may be attributed to the level of experience in engaging with the health care system. In particular, older people in prison may engage more regularly with health care providers in prison due to the reported “greying” and earlier onset of age-related conditions [78]. Similarities were observed in health literacy scores for the remaining scales when comparing younger and older participants. Although the differences were not significant, participants who spoke a language other than English at home reported more difficulty in feeling understood and supported, as well as understanding health information than those who spoke English. It has been reported that people from culturally and linguistically diverse backgrounds face multiple challenges in the multi-health systems [79]. The results of this study may not have found significant differences between non-English and English-speaking participants due to the study design (i.e., the exclusion of people unable to speak English).

### Relationship between health literacy and educational attainment

Higher educational attainment has long been associated with better health outcomes for individuals [80]. People who are incarcerated tend to have lower levels of formal education when compared to general populations [9, 10]. We found those who had completed Year 11 or above scored higher than their less educated counterparts on two scales relating to actively managing their health and understanding health information. This is not surprising as these scales focus on the functional literacy of an individual. Education levels have also been associated with health literacy [25], with anachronistic health literacy tools measuring a unidimensional concept (e.g., functional literacy and/or numeracy). Such tools have limited application when assessing the health literacy of a population [28]. Unexpectedly, our findings did not conform to the previously reported positive associations between higher education levels and health literacy. We found that those who had completed tertiary education (diploma or above) scored higher on only one scale, understanding health information, when compared to those who had not completed post-high school education. Higher scores on this scale may be attributed to the participants’ level of education, confidence and previous exposure to health information. However, participants who reported completing tertiary education scored significantly lower on scale 7, indicating more difficulty navigating the health system, compared to their less educated peers. This finding may be attributed to, at least in part, their exposure to and experience with navigating the prison healthcare system. Lower education levels have previously been shown to be associated with incarceration [81] and type of crime committed [82]. As such, less educated participants may have greater exposure to the prison healthcare system. Due to the context specific nature of health literacy, this may result in health literacy strengths in the prison context compared to their higher educated counterparts, as indicated by a higher HLQ score for scale 7—Navigating the healthcare system. However, this is speculative and requires future research examining associations between education attainment, criminal justice system exposure and access to healthcare to explain this finding. Overall, our findings for educational attainment suggest that, although people with lower educational levels do have different challenges in some health literacy scales, their skills are equal to or better than their more highly educated counterparts in others. Future research is needed to understand the potential mechanisms that might explain the contribution of educational attainment to health literacy.

### Health literacy challenges for those in different prison contexts

People in prison on remand scored lower than their sentenced counterparts with the exception of one health literacy scale (Scale 3—Actively managing my health). Particular difficulties were reported on scales that focused on having sufficient information, and being supported (e.g., socially and by healthcare providers) in prison. These findings, at least in part, could be explained by where people on remand are housed, their access to services and adjustment to their incarceration. People on remand in NSW are usually housed in maximum security prisons [83] which may result in reduced access to the health clinic. Further, people on remand can be restricted in their access to services and programs (i.e. drug and alcohol services) until they are sentenced. This restriction may impact directly or indirectly on their health literacy scores as measured with the HLQ. Due to health literacy being context specific, people who are on remand for the first time may have difficulties in adjusting to the prison health care system (described in the ‘Data Source’ section above) compared to the general community system, which may be reflected in lower HLQ scores. These suggestions are speculative, highlighting the need for further qualitative work to understand the mechanisms underlying the observed difference.

### Strengths and limitations

This is the first study to describe the health literacy of adults in prison using a multi-dimensional health literacy measurement tool in Australia. The HLQ data that has been collected has not only clearly identified areas in which people may need support when navigating the health care system, it has also highlighted health literacy strengths. This study has also shown that a robust health literacy measurement tool, such as the HLQ, can be applied on a large scale. The flow-on effect of this is that it highlights the feasibility for other prison health care providers to investigate the strengths and challenges of this highly vulnerable population group.

The current study has some limitations that need to be acknowledged. Firstly, we present cross-sectional HLQ data; therefore, it is not possible to draw causal conclusions from our results. Secondly, the prison population is fluid in nature. We therefore caution the generalisation of our findings to the larger population. Moreover, in accordance with the informed consent procedures implemented, people who did not have adequate English or capacity to provide informed consent were excluded from participating. Furthermore, those who had not engaged with the health services, or those who were in rural prisons, were not invited to participate. Justice Health NSW provides health care to incarcerated adults across NSW in 36 publicly run correctional centres. Health clinics at 14 metropolitan correctional centres were selected as survey sites, as the study was conducted in the context of the COVID-19 pandemic, restricting researchers’ movements across NSW. Surveys conducted via telehealth were not feasible due to cost and current strain on the system, as well as the written consent process implemented. Exclusion of these cohorts from the sample may result in an over-estimation of the HLQ scores for the NSW prison population. Future research should make efforts to include a more stratified sample including all different population groups in prison. Thirdly, validity evidence for the HLQ in the prison context currently does not exist. The HLQ was designed in a community-based health setting with validity evidence existing for a range of contexts. With this being said, rigorous validity testing of the HLQ in the prison context is required to ensure future decisions are grounded in valid data [31]. Validation is an ongoing process, with validity testing studies requiring both qualitative (e.g., cognitive interviews) and quantitative (e.g., psychometric testing) methods to gather appropriate validity evidence to support the intended use and interpretation of data [84, 85]. Furthermore the researchers undertaking validity testing need to engage with, and listen to, those with lived-experiences to avoid epistemic injustices [31]. That is, harm resulting from undermining the capacity of a certain group of people to participate in the sharing of their knowledge and experiences [31, 86]. To address this limitation (i.e., the lack of validity evidence), a study is currently underway to undertake rigorous validity testing of the HLQ in the prison context using contemporary approaches [84, 87–92]. Cognitive interviews will be used to determine whether participants are answering the items based on their experiences of healthcare in prison and not that of interactions with community-based healthcare providers. Finally, data were not collected regarding the number or type of participant health conditions. We acknowledge the limitation of missing health condition data. Future research is planned to explore the association between health literacy scores and health conditions, the findings of which will be reported separately.

### Implications

Our study findings provide the first insights into the health literacy profile of this population and how it could inform practice (i.e., health care delivery and service provision) and policy (i.e., the rules and regulations that govern the practice) for authorities providing health care in prisons. The findings highlight several areas in which adults in prison and particular participant characteristic groups may require additional support to understand, use and navigate the prison health care system. Our findings suggest that authorities need to consider how health literacy-informed programs could reduce the observed health disparities and inequities of these marginalised populations. A more proactive and responsive approach is needed to be taken by prison and healthcare decision-makers for health literacy; in particular, interventions and actions focusing on long term health benefits [93]. This novel empirical study has created an invaluable evidence base to inform service planning and delivery and influence health policy in the NSW prison context. Further, this evidence base and identification of strengths and challenges makes these findings highly relevant for prison healthcare authorities globally.

## Conclusion

This study shows the usefulness of the HLQ as a health literacy needs assessment in a prison context. Our findings identify health literacy strengths and challenges of adults in NSW metropolitan prisons, adding to the evidence of the tools utility. Compared to the general Australian population, adults in NSW metropolitan prisons had lower HLQ scale scores. Females and people on remand were found to have health literacy challenges across a number of scales when compared to their counterparts. Our findings are highly relevant for the prison context globally and for the provision of equitable health policy and services in particular. These findings highlight the important role health literacy could have in addressing observed health disparities for prison health services.

## Authors’ contributions

The overall study and its design were devised by JB, SK, AL, RZ, PZ and SG. All authors except CS performed the data collection. SG performed the statistical analysis and drafted the manuscript. All authors contributed to the analysis strategy and interpretation of results and had extensive input into the manuscript. All authors approved the final draft.

## Data Availability

The datasets generated during and/or analysed during the current study are available from the corresponding author on reasonable request.

## Abbreviations

CSNSW: Corrective Services NSW
Justice Health NSW: Justice Health and Forensic Mental Health Network
HLQ: Health Literacy Questionnaire
ES: Effect Size
95% CI: 95% Confidence Interval
SD: Standard Deviation
